# Quercetin- and caffeic acid-functionalized chitosan-capped colloidal silver nanoparticles: one-pot synthesis, characterization, and anticancer and antibacterial activities

**DOI:** 10.3762/bjnano.14.31

**Published:** 2023-03-20

**Authors:** Akif Hakan Kurt, Elif Berna Olutas, Fatma Avcioglu, Hamza Karakuş, Mehmet Ali Sungur, Cansu Kara Oztabag, Muhammet Yıldırım

**Affiliations:** 1 Department of Medicinal Pharmacology, Faculty of Medicine, Bolu Abant Izzet Baysal University, 14030 Bolu, Türkiye; 2 Department of Chemistry, Faculty of Arts and Sciences, Bolu Abant Izzet Baysal University, 14030 Bolu, Türkiye; 3 Department of Medical Microbiology, Faculty of Medicine, Bolu Abant Izzet Baysal University, 14030 Bolu, Türkiye; 4 Technology Transfer Application and Research Center, Bolu Abant Izzet Baysal University, 14030 Bolu, Türkiye; 5 Department of Biostatistics and Medical Informatics, Faculty of Medicine, Duzce University, 81620 Duzce, Türkiye; 6 Department of Interdisciplinary Neuroscience, Graduate Education Institute, Bolu Abant Izzet Baysal University, 14030 Bolu, Türkiye

**Keywords:** Ag NPs, anticancer and antibacterial effects, caffeic acid, chitosan, one-pot synthesis, quercetin, U-118 MG and ARPE-19 cells

## Abstract

The presented study comprises the one-pot synthesis and the characterization of quercetin- and caffeic acid-functionalized chitosan-capped colloidal silver nanoparticles (Ch/Q- and Ch/CA-Ag NPs), and their antibacterial and anticancer activities. The formation of Ch/Q- and Ch/CA-Ag NPs has been confirmed by ultraviolet–visible (UV–vis) spectroscopy, Fourier-transform infrared (FTIR) spectroscopy, and transmission electron microscopy (TEM). The characteristic surface plasmon resonance (SPR) absorption band has been found at 417 and 424 nm for Ch/Q- and Ch/CA-Ag NPs, respectively. The formation of a chitosan shell comprising quercetin and caffeic acid, which surround the colloidal core Ag NPs, was confirmed by UV–vis, and FTIR analyses, and monitored by TEM microscopy. The size of nanoparticles has been determined as 11.2 and 10.3 nm for Ch/Q- and Ch/CA-Ag, respectively. The anticancer activity of Ch/Q- and Ch/CA-Ag NPs has been evaluated against U-118 MG (human glioblastoma) and ARPE-19 (human retinal pigment epithelium) cells. Both NPs showed anticancer activity, but Ch/Q-Ag NPs seemed to be more effective on cancer cell lines (U-118 MG) in comparison to healthy ones (ARPE-19). Furthermore, the antibacterial activity of Ch/Q- and Ch/CA-Ag NPs against Gram-negative (*P. aeruginosa* and *E. coli*) and Gram-positive (*S. aureus* and *S. epidermidis*) bacteria was determined, and dose-dependent antibacterial effects were found.

## Introduction

In recent years, nanoscale studies have become an important research area thanks to the ever increasing means of synthesis, characterization, and application of nanoscale materials (1 to 100 nm). Progress and development in nanotechnology have started to make a difference in various areas, especially in medicine [1]. Nanomaterials, especially metallic nanomaterials, show good antibacterial and antimicrobial properties due to their physical and chemical properties and are, thus, an alternative approach to antibiotics [2]. In addition, low bioavailability and water solubility problems are the biggest problems in the development of new drugs [3]. One of the approaches to overcome these problems is nanoparticle carriers. These carriers are advantageous because their high surface area and good adhesion to biological surfaces increase solubility and dissolution rate [4].

The Gram-positive *Staphylococcus aureus* (*S. aureus*) and *Staphylococcus epidermidis* (*S. epidermidis*) and the Gram-negative *Escherichia coli* (*E. coli*) and *Pseudomonas aeruginosa* (*P. aeruginosa*) bacteria cause various infections [5]. These infections, formerly known as nosocomial infections, are now referred to as healthcare-associated infections and adversely affect both economies of countries and patient survival rates. The World Health Organization (WHO) has predicted that there will be approximately 10 million deaths due to antibiotic resistance by the year 2050. Therefore, antibiotic resistance, which has received great attention as an important problem of the modern era, encourages the discovery of new antimicrobial and antibacterial agents [6–7].

Glioblastoma multiforme (GBM, grade IV) with a low survival rate is the most commonly malignant and invasive tumor of the central nervous system and it is resistant to conventional treatments. This resistance is mostly due to the blood–brain barrier, which is the most important obstacle to drug distribution. Since nanoparticles can penetrate through the blood–brain barrier, they are a preferred medicine in brain and nervous system diseases. In glioblastoma multiforme treatment, nanoparticles have an important place to overcome the physiological limitations and to improve therapeutic efficiency [8–9].

Chitosan is a natural polysaccharide biopolymer of different molecular weights bearing amino and hydroxy functional groups [10]. It is the main component of shellfish such as crabs, and shrimps. It is also found in the skeleton of insects and the structure of cell walls of fungi [11–12]. Chitosan, which was defined as an antimicrobial agent for the first time by Allan and Hadwiger, exhibits broad-spectrum antimicrobial activity [13–14]. In the last decade, chitosan has raised great interest in the preparation of nanoparticles owing to its biodegradable, biocompatible, and non-toxic properties, as well as the free amino groups, which are very suitable to produce strong complexes with metals [15–16].

Quercetin (3,3',4',5,7-pentahydroxyflavone) is a flavonoid present in plants such as onions, soybeans, lettuce, apples, red grapes, broccoli, and tomatoes [17]. It is also a naturally forming polar auxin transport inhibitor [18]. Various biological and pharmacological properties of quercetin have been reported, including antiviral, antibacterial, antimicrobial, anti-inflammatory, antidiabetic, antitumor, antioxidant, lipid-lowering, and wound healing properties [19–23]. Although many studies have been conducted in recent years regarding the synthesis of new structures with quercetin and their biological properties [24–28], there is continued attention due to the unique properties of quercetin.

Caffeic acid (3,4-dihydroxycinnamic acid) is a natural phenolic acid with high antioxidant capacity due to the catechol group. It is present in, for example, fruits, olive oil, green tea, coffee, vegetables, and white wine [29–30]. In addition, caffeic acid has also been reported to have antiviral, anticoagulant, anti-inflammatory, antibacterial, and anticancer activities [31–34].

Silver nanoparticles are an important example of different types of nanomaterials (copper, zinc, titanium, magnesium, gold, and alginate) that have been proven to be effective against bacteria and viruses [6,35]. Toxicologists extensively studied silver nanoparticles. Despite studies suggesting a toxicity of silver nanoparticles, this issue is still unresolved [36]. However, silver nanoparticles have become more popular in recent years due to their unique properties, low cost, and low cytotoxicity [37–39]. Hybrid structures containing silver played an important role in the development of strong antibacterial agents and do not cause drug resistance problems due to their broad-spectrum antibacterial action [40]. These features led to a wide range of uses such as antibacterial and antimicrobial agents, healthcare-related products, medical device coatings, anticancer agents, optical sensors, anti-inflammatory agents, biocatalysts, cosmetics, and biosensors [41–45]. The combination of polymer-coated metal nanoparticles, especially chitosan–silver nanocomposites, which yield a new type of nanoparticles, has raised more attention regarding eco-friendly properties and applications in nanomedicine and environmental remediation. Syntheses of chitosan, silver, and quercetin alone or in binary combinations, that is, chitosan–silver [46–47], chitosan–quercetin [48–49], and silver–quercetin nanoparticles [50–51], are well known. For instance, chitosan–silver nanocomposites, which have been synthesized through bio-inspired and photosynthesis, exhibited significant antioxidant, photocatalytic, and antibacterial activities, in contrast to pure silver nanoparticles [46,52–55].

To the best of our knowledge, there is no scientific study on the ternary synthesis of quercetin and caffeic acid-functionalized chitosan-capped colloidal silver nanoparticles (Ch/Q- and Ch/CA-Ag NPs) in a one-pot manner. Here, we report for the first time the one-pot synthesis of Ch/Q- and Ch/CA-Ag NPs and their biological properties (anticancer and antibacterial). Ag NPs have been prepared and characterized by UV–vis, FTIR, and TEM measurements. In another study of ours, Lomustine, a common drug against glioblastoma cancer, was applied at a high dose (500 µM) for 24 h. It decreased cell viability by 75% in glioblastoma cells and by 25% in non-cancerous cells (data not shown). From this, it can be concluded that the selected cancer drug is highly specific to the cancer cells [56]. Therefore, human glioblastoma (U-118 MG) cell lines were used to investigate the specificity of nanoparticles to the glioblastoma multiforme cancer cells in the current study. ARPE19 cells as a part of the central nervous system were also used as the non-cancer cell (control). In addition, the antibacterial activity of the synthesized Ag NPs was tested against some Gram-positive and Gram-negative bacteria. The prepared ternary systems (Ch/Q- and Ch/CA-Ag NPs) are expected to have better cytotoxic properties than binary systems (chitosan–silver and quercetin–silver), which showed moderate cytotoxicity in vitro at higher doses against different cancer cell lines (MCF-7 and HepG2) [57–58].

## Materials and Methods

### Chemicals

**Figure 1:**
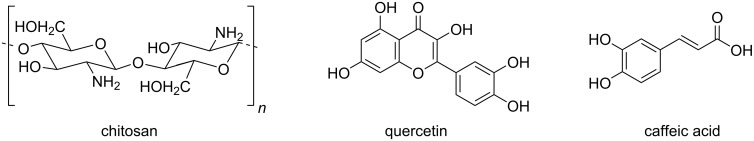
Chemical structures of chitosan, quercetin, and caffeic acid, respectively.

Chitosan (medium molecular weight: 190,000–310,000 Da based on viscosity; 75–85% deacetylated; viscosity: 200–800 cP, that is 1 wt % in 1% acetic acid at 25 °C), sodium borohydride (NaBH_4_, ≥98.0%), caffeic acid (≥98.0%), and quercetin (≥95%) were purchased from Sigma-Aldrich. Silver nitrate (AgNO_3_, ≥99.8%) was obtained from ISOLAB. Dimethyl sulfoxide (DMSO, ≥99.0%), glacial acetic acid (CH_3_COOH), anhydrous aluminium chloride (AlCl_3_, ≥98.0%), and Folin–Ciocalteau’s phenol reagent (2 N) were purchased from Merck. Water was purified through a Milli-Q water purification system. Figure 1 shows the chemical structures of chitosan, quercetin, and caffeic acid.

### Synthesis of silver nanoparticles

The chitosan-capped colloidal silver nanoparticles (Ch-Ag NPs) were synthesized by reducing AgNO_3_ with NaBH_4_ in the presence of chitosan as described in [59–60] with slight modifications. A solution of 0.1% chitosan (w/v) was prepared in 1% acetic acid solution under stirring at room temperature for about 24 h. Then, the chitosan solution was placed in an ice bath. To this solution, 1 mL of 50 mM AgNO_3_ aqueous solution was added to obtain the final concentration of 0.5 mM. After that, 1 mL of freshly prepared 0.5 M NaBH_4_ was swiftly introduced and this mixture was stirred in an ice bath for 150 min. During the reaction, the color of the solution changed to clear yellow, brown, and brownish yellow. Finally, the solution was allowed for aging at room temperature before the purification step. The Ch-Ag NPs were cleaned with 1% acetic acid solution to remove unreacted chitosan by ultracentrifuging (Hitachi, Himac CP100 wx) at 40,000 rpm for 30 min. The collected Ch-Ag NPs were dispersed in distilled water and filtered through a 0.22 µm syringe filter (Sartorius, NY). The resulting Ch-Ag NPs were kept in a refrigerator at 4 °C in the dark for further use.

Quercetin- and caffeic acid-functionalized chitosan-capped colloidal silver nanoparticles (Ch/Q- and Ch/CA-Ag NPs) were also synthesized following the above procedure. In this case, 0.1% chitosan in 1% acetic acid solution was prepared to contain 0.5 mM of quercetin (or caffeic acid) in DMSO. All other procedures were applied in the same way. Ch/Q- and Ch/CA-Ag NPs were also stored in a refrigerator at 4 °C in the dark for further use.

### Characterization of silver nanoparticles

The absorption spectra of quercetin, caffeic acid, chitosan, Ch-, Ch/Q-, and Ch/CA-Ag NPs were recorded on a UV–vis double-beam spectrophotometer (Hitachi U-2900) at wavelengths of 200 to 800 nm at 1.0 nm intervals with a quartz cell of 1.0 cm light path. Fourier-transform infrared (FTIR) spectra of quercetin, caffeic acid, chitosan, Ch/Q-, and Ch/CA-Ag NPs were monitored using a Perkin Elmer Spectrum Two FTIR-ATR spectrophotometer in the range of 4000–400 cm^−1^.

Transmission electron microscopy (TEM) measurements were performed with a JEOL JEM-2100 electron microscope operating at 200 kV. Small volumes of Ch/Q- and Ch/CA-Ag NPs were placed on carbon-coated copper grids and allowed to evaporate at room temperature. For negative staining, a drop of freshly prepared 2% uranyl acetate solution was dripped on the copper grid, and excess liquid is removed by a piece of paper after 2 min.

Zeta potential measurements were carried out using a Malvern Zetasizer Nano–Z instrument at 25 ± 0.1 °C. The data was taken as the average of three independent measurements of 10 runs.

Inductively coupled plasma optical emission spectroscopy (ICP-OES) measurements were carried out using Perkin Elmer Avio 220 Max to determine the concentration of the synthesized Ch/Q- and Ch/CA-Ag NPs stock solutions. The Ag concentrations of Ch/Q- and Ch/CA-Ag NPs stock solutions were 82.805 and 136.303 mg/L, respectively.

### Determination of amount of quercetin using total flavonoid content

The standard quercetin solutions at certain concentrations (50–200 μg/mL) were prepared in DMSO. Each standard solution (100 µL) was then diluted with distilled water (1.8 mL) and mixed with a 2% aqueous solution of anhydrous AlCl_3_ (100 µL). The obtained mixtures were vortexed. After incubation for 10 min at ambient temperature, absorbances of the mixtures were measured between 200 and 800 nm wavelengths using a UV–vis double-beam spectrophotometer (Hitachi U-2900). The same method was also applied to the sample of Ch/Q-Ag NPs. Unlike the standards, the Ch/Q-Ag sample was centrifuged, and the absorbance of the supernatant was recorded at 433 nm. Distilled water with a quartz cell of 1.0 cm light path was used as a reference for the measurements.

### Determination of amount of caffeic acid using total phenolic content

Standard caffeic acid solutions at concentrations of 50–200 μg/mL were prepared in DMSO. Standard caffeic acid solution (50 µL) was mixed with distilled water (1.45 mL) followed by 2 N Folin–Ciocalteau’s reagent (125 µL). The obtained mixtures were vortexed and incubated at ambient temperature for 10 min. Then, to these mixtures, an aqueous Na_2_CO_3_ solution (20% w/v, 375 µL) was added. All the mixtures were vortexed and incubated at ambient temperature for 1 h. Afterward, the absorbances of these mixtures were measured between 200 and 800 nm using a UV–vis double-beam spectrophotometer (Hitachi U-2900). The same method was also applied to the sample of Ch/CA-Ag NPs. The absorbance of the blue color solution of the Ch/CA-Ag sample was recorded at 762 nm. During the measurements, distilled water was used as a reference.

### Cell culture

Human brain glioma (U-118 MG) and human retinal pigment epithelium (ARPE-19) cell lines were purchased from ATCC (NY, USA) and cultured in 10% (v/v) FBS (Gibco; Thermo Scientific, USA) and 1% (v/v) penicillin/streptomycin (Gibco; Thermo Scientific, USA) containing high-glucose DMEM (Gibco; Thermo Scientific, USA) in an incubator at 37 °C with 5% CO_2_ pressure.

### Cell viability assay (XTT)

The cells were planted at approximately 10,000/100 µL per well in 96-well plates. To reveal the dose–time response, the cells were treated for 24 h in a medium with five different concentrations of Ch/Q- and Ch/CA-Ag NPs in dilutions of 1/1, 1/2, 1/3, 1/4, and 1/5 (v/v), where DMEM without phenol red was used for dilution of Ag NPs. Since the NPs were dispersed in distilled water, distilled water without NPs was used as control group. After 24 h of incubation, the medium containing the Ag NPs was removed and the cells were washed at least twice with 100 μL of DMEM without phenol red. The cell viability was evaluated with XTT (3'-[1-phenylaminocarbonyl-3,4-tetrazolium]bis(4-methoxy-6-nitro)benzenesulfonic acid hydrate, Cell Proliferation Kit II test (Roche). XTT solution was added to each plate at 50 µL and was incubated for 4 h at 37 °C. Then, the absorbance (optical density, OD) of the solution in each plate was read at 450 nm by a spectrophotometer (BiotekELx800, Winooski, VT, USA).

### Statistical analysis

Statistical analyses were done using the IBM SPSS v.22 statistical packages. Normality assumption was examined with the Shapiro–Wilk test. A one-way ANOVA followed by Tukey HSD and Dunnett *t* post hoc tests were used to compare groups. The Kruskal–Wallis test followed by the Dunn test as post hoc test was used to compare groups for variables that did not meet the normality assumption. Descriptive statistics were presented as mean and standard deviation, or median (min–max), as appropriate. A *p*-value of 0.05 was considered a level of statistical significance.

### Antibacterial activity

Standard strains of *S. aureus* (ATCC 25923), *S. epidermidis* (ATCC 12228), *P. aeruginosa* (ATCC 27853), and *E. coli* (ATCC 8739), which are generally opportunistic pathogens, were used in the study. The antibacterial activity of diluted nanoparticle solutions was investigated by the agar disc diffusion method. The antibacterial activity of NPs was compared to the inhibition zone diameters of control antibiotics. Amikacin antibiotic was used as a positive control for *P. aeruginosa*, and ampicillin antibiotic was used as a positive control for *E. coli*, *S. aureus*, and *S. epidermidis*. The susceptibility zone diameters were *S* ≥ 15 mm for amikacin against *P. aeruginosa*, *S* ≥ 14 mm for ampicillin against *E. coli*, *S* ≥ 18 mm for ampicillin against *S. aureus*, and *S* ≥ 18 mm for ampicillin against *S. epidermidis*, measured according to the European committee on antimicrobial susceptibility testing (EUCAST) [61–62]. To resuscitate standard bacterial strains, 5% sheep blood agar and eosin methylene blue (EMB) media were used. The inoculated plates were kept in an oven at 35 ± 2 °C for 18–24 h. Sterile Müller Hinton broth fluid tubes were prepared in 0.5 McFarland turbidity standard for each bacterial strain. The bacteria were inoculated on Müller Hinton agar medium. Under sterile conditions, blank discs (6 mm) were impregnated with 10 µL of diluted Ch/Q- and Ch/CA-Ag NP solutions, prepared from a stock solution in dilutions of 1/1, 1/3, and 1/5 (v/v), and these discs were placed on Müller Hinton agar media prepared for each bacterial strain. Then the inoculated plates were kept in an oven at 35 ± 2 °C for 18–24 h. The diameters of the inhibition zones formed around the discs in the medium were measured with millimetric rulers. This process was repeated three times for each antibacterial solution. A statistically average value was calculated from the obtained results.

## Results and Discussion

**Figure 2:**
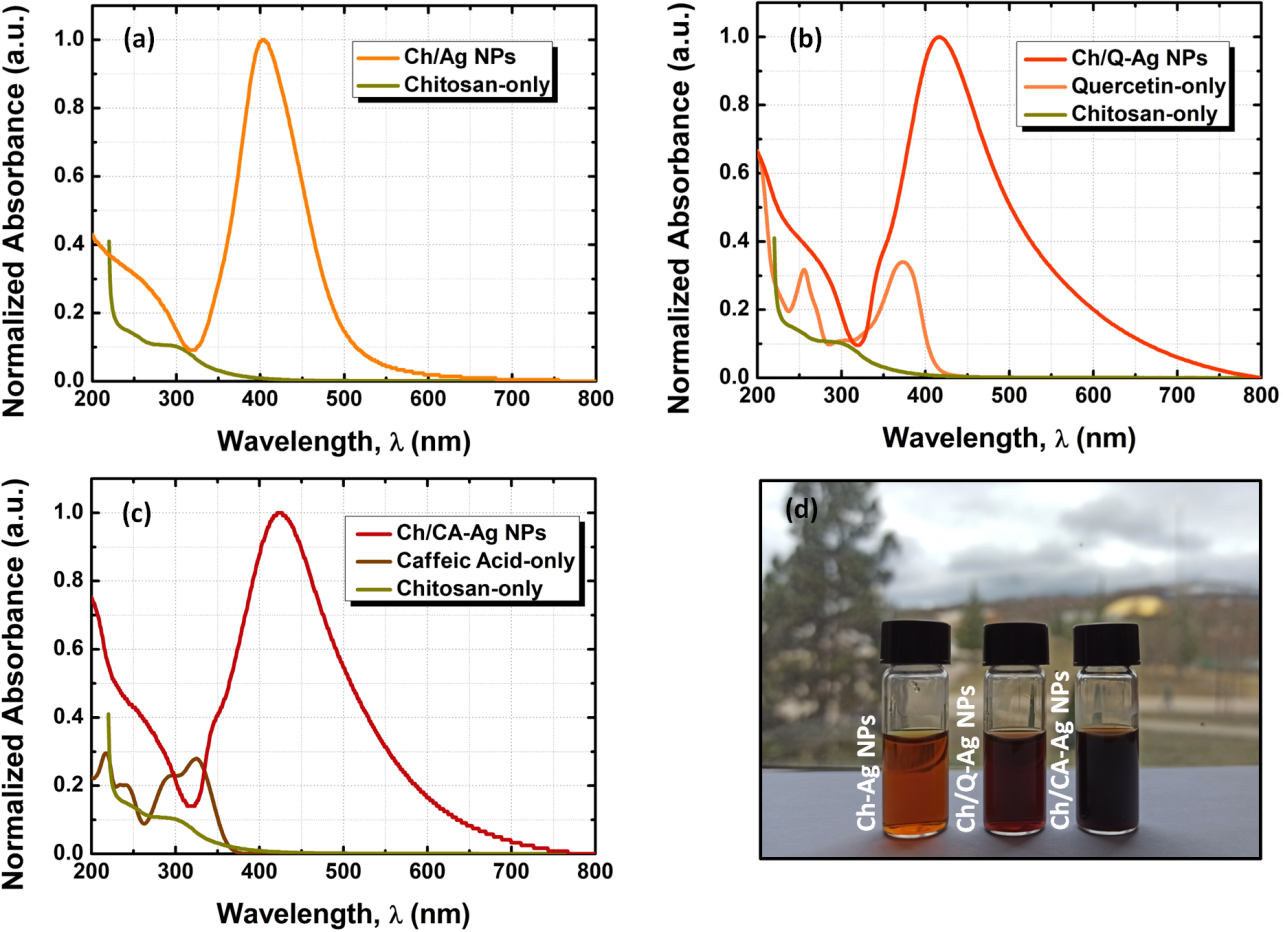
UV–vis absorption spectra of (a) chitosan-only (Ch-), (b) chitosan/quercetin (Ch/Q-), and (c) chitosan/caffeic acid (Ch/CA-) capped Ag NPs. (d) The picture of the synthesized Ch-, Ch/Q-, and Ch/CA-Ag NPs under daylight.

The characterization of the synthesized silver nanoparticles (Ag NPs) was carried out by using UV–vis absorption spectroscopy at ambient temperature. Figure 2 shows the UV–vis absorption measurement results of different Ag NPs, which are either covered by only chitosan (Ch-Ag NPs) or chitosan with quercetin and caffeic acid as co-capping agents (Ch/Q- and Ch/CA-Ag NPs), and pictures of the nanoparticle solutions under daylight.

As seen in Figure 2a, the absorption curve of the Ch-Ag NPs ends at around 600 nm, and the maximum for the characteristic surface plasmon resonance (SPR) absorption peak, which is due to the collective oscillation of free surface electrons in resonance with the electric field component of incoming photons, is located at 404 nm. However, the evolution of the absorption curve exhibits changes after the introduction of quercetin (or caffeic acid) into the structure of chitosan surrounding the Ag NPs. For instance, it is seen in Figure 2b and Figure 2c that the SPR peaks shift to 417 and 424 nm for Ch/Q- and Ch/CA-Ag NPs, respectively, while their absorption curves broaden to the near-IR region of the spectrum. The redshift of the SPR peak implies an increase in the size of Ag NPs. In addition, the broadening of the spectrum can be explained by the increasing size and shape distribution arising from excess co-capping agents, which are present in the reaction medium during the reduction of Ag ions. The broadening in the absorption spectra is also reflected in the color of nanoparticle solutions (see Figure 2d). Compared to the reference sample (i.e., Ch-Ag NPs), new absorption shoulders appeared at the higher energy side of the spectra (i.e., at about 200 and 345 nm) in the modified chitosan structure with quercetin or caffeic acid. These new shoulders indicate the presence of quercetin and caffeic acid in the modified chitosan surrounding Ag NPs. As seen in Figure 2b, the peaks at around 200, 250, and 370 nm in the absorption curve of quercetin match well with the shoulders that appear due to the contribution of quercetin in the absorption spectrum of Ch/Q-Ag NPs. A similar behavior can also be seen in the absorption spectrum of the Ch/CA-Ag NPs with a small shift compared to that of caffeic acid. The absorption peak at around 325 nm of the caffeic acid does not fully match with the shoulder appearing at the higher energy side of the SPR peak of the Ch/CA-Ag NPs while its absorption tail overlaps with that of the SPR peak. These partial matches in the absorption features might be due to the molecular interactions between caffeic acid and chitosan/silver nanostructures [63].

**Figure 3:**
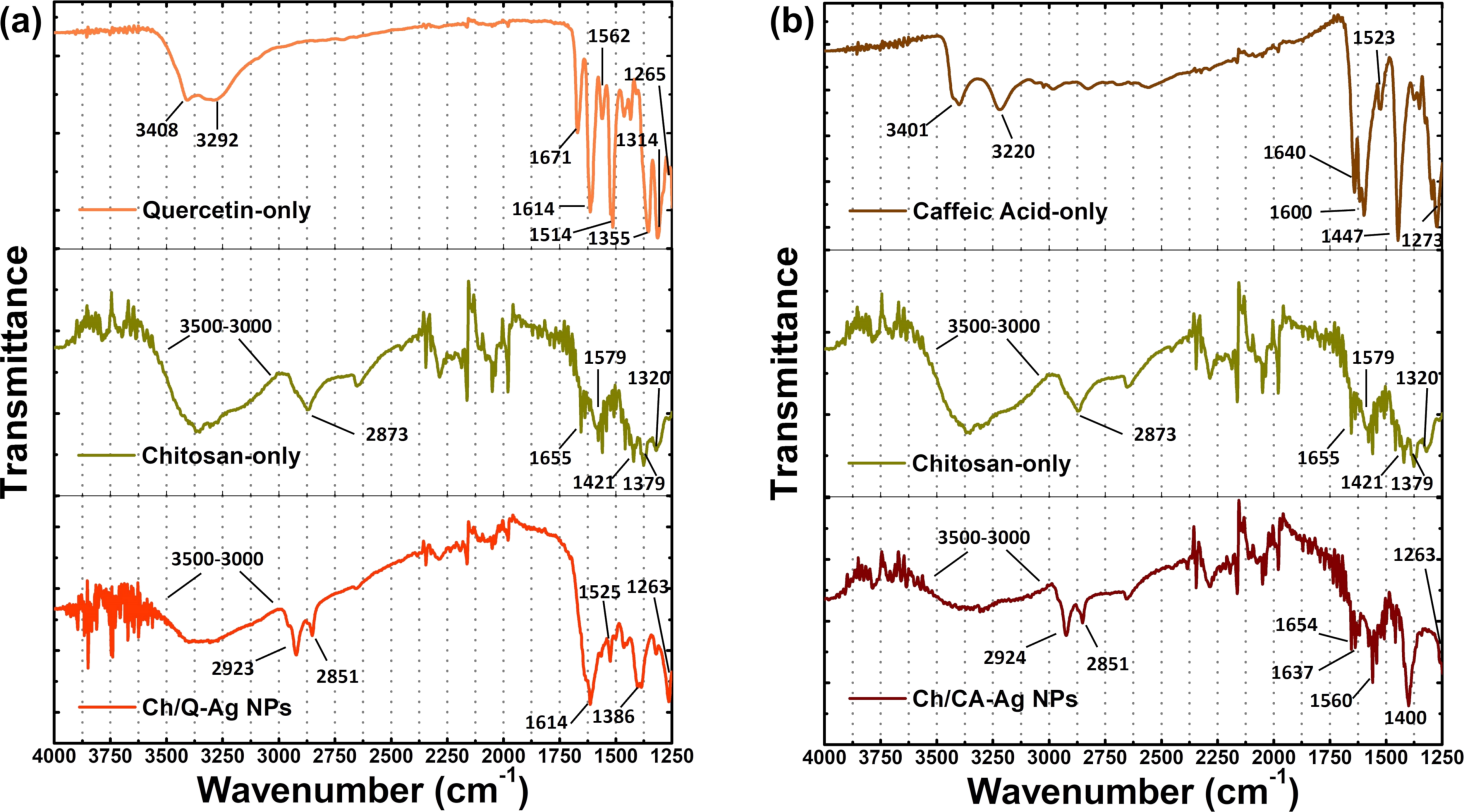
FTIR spectra of (a) the chitosan/quercetin (Ch/Q-)-, and (b) the chitosan/caffeic acid (Ch/CA-)-capped Ag NPs, including the spectra of chitosan, quercetin, and caffeic acid used in the synthesis of the NPs.

FTIR spectroscopy measurements were performed to confirm the formation of Ch/Q- and Ch/CA-Ag NPs and to obtain information about the interaction in the synthesized materials using characteristic bands of functional groups. Figure 3 depicts the FTIR spectra of the synthesized Ag NPs and the pure materials (quercetin, caffeic acid, and chitosan) used in the structure of the shell layers.

In the FTIR spectrum of pure quercetin (Figure 3a) the bands observed at about 3408 and 3292 cm^−1^ correspond to phenolic O–H stretching [64]. The peak at 1671 cm^−1^ stems from the stretching of the C=O carbonyl functional group. The characteristic C=C aromatic ring stretching bands were observed at 1614, 1562, and 1514 cm^−1^. The peaks at 1355, 1314, and 1265 cm^−1^ were attributed to aromatic C–H and O–H bending vibrations in quercetin. The FTIR spectrum of pure caffeic acid (Figure 3b) shows intense bands at 3401 and 3220 cm^−1^ indicating O–H stretching. The very strong band at 1640 cm^−1^ corresponds to a conjugated C=O stretching vibration. The characteristic bands at 1600, 1523, and 1447 cm^−1^ were assigned to both aromatic and olefinic C=C stretching modes. The strong band at 1273 cm^−1^ is due to the in-plane bending of C–H bonds. The FTIR spectrum of pure chitosan exhibit overlapping O–H and N–H stretching bands showing a broad band between 3500 and 3000 cm^−1^. C–H stretching is evident at 2873 cm^−1^. The bands at 1655, 1579, and 1320 cm^−1^ were assigned to C=O stretching (amide I), N–H bending (amide II), and C–N stretching (amide III) modes, respectively, due to the presence of residual *N*-acetyl groups [63,65]. The peaks at 1421 and 1379 cm^−1^ are due to C–H bending and symmetrical deformation modes. In the FTIR spectra of Ch/Q- and Ch/CA-Ag NPs, a decrease in the intensity of broad bands at around 3500–3000 cm^−1^ were attributed to the hydrogen bonding interactions between amino groups of chitosan and phenol groups of quercetin and caffeic acid [63,66]. Moreover, it was observed that the position and shape of some peaks, such as 1671 and 1514 cm^−1^ for quercetin, 1421 and 1379 cm^−1^ for chitosan, and 1640 and 1447 cm^−1^ for caffeic acid, also changed due to the formation of links between quercetin–chitosan–Ag and caffeic acid–chitosan–Ag in the synthesized NPs.

**Figure 4:**
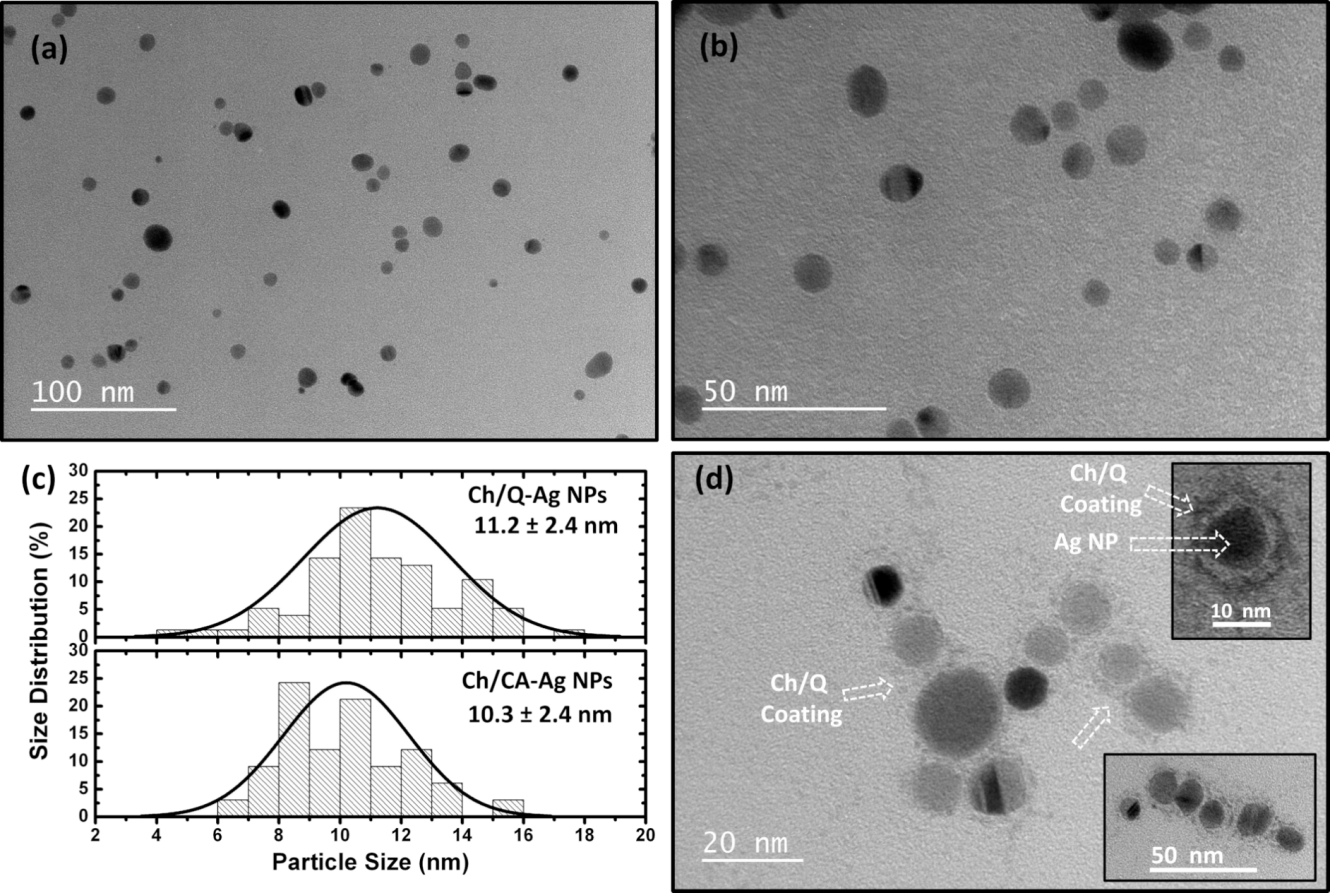
TEM images of (a) the chitosan/quercetin- (Ch/Q-) and (b) the chitosan/caffeic acid (Ch/CA-)-capped Ag NPs. (c) Size distribution analysis of the synthesized Ch/Q- and Ch/CA-Ag NPs. (d) TEM image of the Ch/Q-Ag NPs taken by the negative staining method using uranyl acetate. The insets of (d) show the images of the stained shell structure of the Ch/Q-Ag NPs.

TEM was utilized to investigate the size and morphological features of the synthesized Ag NPs. Figure 4 shows TEM images of Ch/Q- and Ch/CA-Ag NPs. As can be seen from the TEM images in Figure 4a and Figure 4b, the Ag NPs covered by chitosan layers comprising quercetin or caffeic acid have mostly spherical shapes. However, both samples also include nanostructures with different shapes, such as rods and triangles, in smaller numbers than the spherical particles. This also resulted in broadening the absorption curve to the lower-energy side (i.e., longer wavelength) of the UV–vis spectrum for both samples (see Figure 2b,c). The size analysis of both Ch/Q- and Ch/CA-Ag NPs was performed using ImageJ software, and their average size distributions were determined using Gaussian distribution fitting. Figure 4c indicates the size distribution of both samples. The average particle size of the Ch/Q-Ag NPs was calculated as 11.2 ± 2.4 nm, whereas it was found to be 10.3 ± 2.4 nm for the Ch/CA-Ag NPs. To visualize the organic shell structure that covers the Ag NPs, which is difficult to display in TEM due to energetic electron bombardment causing burn, negative staining using uranyl acetate was performed. Figure 4d shows the TEM image of the Ch/Q-Ag NPs exposed by negative staining. It is seen that the chitosan/quercetin shell structure uniformly covers the core Ag NPs (see insets of Figure 4d).

**Figure 5:**
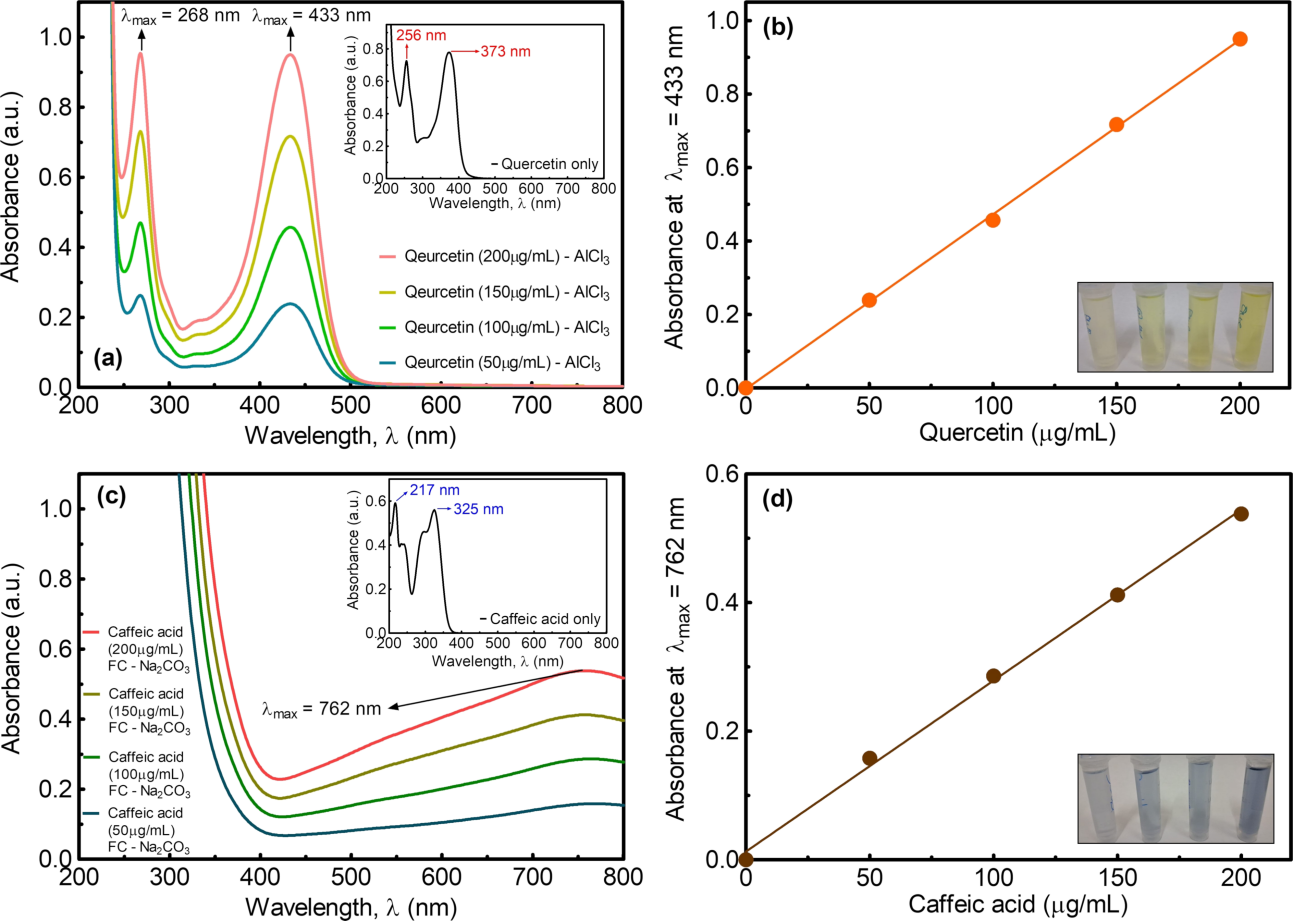
(a) UV–vis absorption spectra of the quercetin–AlCl_3_ complex and quercetin only (inset figure). (b) A calibration curve (*r*^2^ = 0.9994) was obtained for the quercetin–AlCl_3_ complex at λ_max_ = 433 nm. (c) UV–vis absorption spectra of caffeic acid–FC–Na_2_CO_3_ mixture and caffeic acid only (inset figure). (d) A calibration curve (*r*^2^ = 0.9977) was obtained for the caffeic acid–FC–Na_2_CO_3_ mixtures at λ_max_ = 762 nm.

In addition, the amount of the capping agents (quercetin and caffeic acid) that may be of high importance for potential applications of NPs has been determined. As known, quercetin is one of the main reagents used as a reference standard for the quantification of flavonoids in a sample [67]. The total flavonoid content of a sample can be determined by the AlCl_3_ colorimetric method using a UV–vis spectrophotometer [67–69]. In this method, quercetin, which gives a light yellow solution, forms a quercetin–AlCl_3_ complex after mixing with aqueous AlCl_3_ solution, yielding an intensely yellow solution [67]. Because of the formation of the quercetin–AlCl_3_ complex, a red shift is observed in the absorption peaks of the quercetin. Since the absorption band observed between 400 and 450 nm does not have any overlap with pure quercetin, it is generally used to draw the calibration curve. Considering that the total amount of flavonoids in pure quercetin is proportional to the quercetin concentration, it was aimed to reveal the amount of quercetin in the Ch/Q-Ag NPs with the same method [70]. In the absorption spectrum in Figure 5a (inset graph), two absorption bands were observed at 256 and 373 nm for pure quercetin, while the quercetin–AlCl_3_ complex gave absorption bands at 268 and 433 nm (Figure 5a). The observed absorption band at 433 nm is the most suitable region to determine the total flavonoid content. Therefore, the wavelength of maximum absorbance (λ_max_) at 433 nm was chosen to obtain the calibration curve (Figure 5b). From this calibration curve and the absorbance of the Ch/Q-Ag sample measured at 433 nm, the amount of quercetin in the Ch/Q-Ag sample was determined to be approximately 31.0 ± 0.8 μg/mL. Similarly, the amount of caffeic acid, which is a phenolic compound, in the Ch/CA-Ag NPs has been determined from the phenolic content assay. To this end, the Folin–Ciocalteau (FC) assay, which is a method frequently used in the literature to determine the phenolic content of the samples, has been used [67–70]. Here, because of the reaction that takes place in the basic environment, caffeic acid is oxidized, while the FC reagent is reduced, giving the solution a blue color (λ ≈ 760 nm), that is, a colorimetric reaction [67,69,71–72]. This reaction can also be monitored by a UV–vis spectrophotometer, and the observed broad absorption band varies depending on the concentration of phenolics. Figure 5c shows the absorption spectra of pure caffeic acid (inset graph) and caffeic acid–FC–Na_2_CO_3_ mixtures. The calibration curve was obtained using absorbance values at 762 nm (Figure 5d). Since the phenolic content will be proportional to the amount of caffeic acid present in the medium, the amount of caffeic acid has been determined from the calibration curve and the absorbance of the Ch/CA-Ag sample measured at 762 nm for the synthesized Ch/CA-Ag sample to be 28.8 ± 0.4 μg/mL.

**Figure 6:**
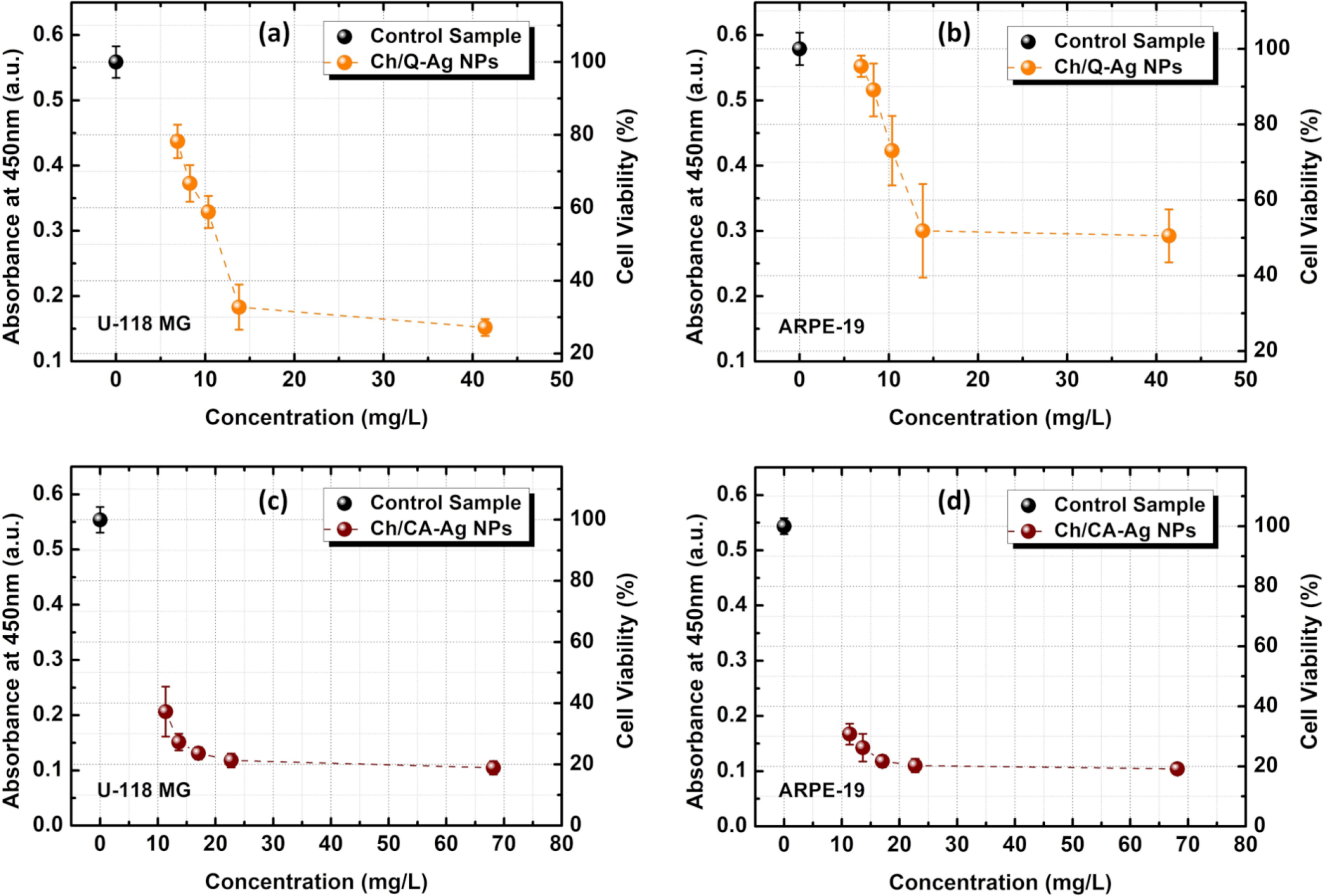
Concentration (dose)-dependent viability of U-118 MG and ARPE-19 cell lines for (a, b) the chitosan/quercetin (Ch/Q-)-, and (c, d) the chitosan/caffeic acid (Ch/CA-)-capped Ag NPs, respectively.

Anticancer activity studies were also conducted for the synthesized Ch/Q- and Ch/CA-Ag NPs using XTT assay. As known, XTT and MTT, tetrazolium salts, are used to evaluate cell viability by a colorimetric method based on their reduction to colored formazan compounds by living cells. Actively respiring cells convert water-soluble XTT to a water-soluble, orange-colored formazan product, while water-soluble MTT converts to an insoluble purple formazan. Therefore, MTT requires solubilization of insoluble formazan to determine its concentration by absorbance, whereas XTT does not require any solubilization. Therefore, the use of XTT is an excellent solution for the quantification of cells and their viability, as it greatly simplifies the procedure for measuring proliferation over MTT, reduces assay time, and increases the sensitivity of the assay [73]. In this study, the dose-dependent cell viabilities of human brain glioblastoma (U-118 MG) and human retinal pigment epithelium (ARPE-19) cell lines after administration of Ch/Q- and Ch/CA-Ag NPs were measured using XTT assay (Figure 6). Another consideration in determining cell viability is the wavelength chosen for absorbance measurements of XTT (450 nm). Since XTT is in the same region with Ag NPs that can absorb (or scatter) light at this wavelength (450 nm), necessary washings were made after incubation to prevent misleading effects of Ag NPs on absorbance, and then the XTT test was applied (see section “Cell viability assay (XTT)”). Thus, the absorbance contribution that may arise from Ag NPs is eliminated. As seen in Figure 6a and Figure 6b, the absorbance values obtained for Ch/Q-Ag NPs after XTT application in U-118 MG and ARPE-19 cell lines at 6.9 mg/L (1/5 dilution) were approximately 0.45 and 0.55, respectively. If Ch/Q-Ag NPs had any effect on the absorbance measured at 450 nm, the absorbance at the highest dose of 41.4 mg/L (1/1 dilution) would be expected to be much higher than the other measured doses (1/2, 1/3, 1/4, and 1/5 dilutions). In addition, after the application of Ch/Q-Ag NPs to the U-118 MG cell line, it was observed from the microscope images that the cell viability decreased as the dose increased compared to the control (see Supporting Information File 1, Figure S1). Especially at low doses (Supporting Information File 1, Figure S1b), cell viability seemed to be very close to the control (Supporting Information File 1, Figure S1a). As a result, it is concluded that there is no interference of Ag NPs with XTT, so the absorbance values obtained are specific results showing only cell viability.

As seen in Figure 6, the cell viability values for all Ch/Q- and Ch/CA-Ag NPs doses were lower than those of the control group according to the Dunnett t post hoc test results (all p-values were below 0.001 for all doses). When Ch/Q- and Ch/CA-Ag NPs were used in the U-118 MG cell line, the highest cell viabilities were found at 6.9 and 11.4 mg/L Ag concentration (1/5 dilution) for quercetin and caffeic acid, respectively (Figure 6a and Figure 6c). For Ch/Q-Ag NPs, the lowest cell viability in the U-118 MG cell line was seen at 41.4 mg/L (1/1 dilution) and 13.8 mg/L (1/2 dilution) with *p* = 0.176. Also, the cell viability for Ch/Q-Ag NPs in U-118 MG was 78% at 6.9 mg/L (1/5 dilution), whereas it reduced to 27% at 41.4 mg/L (1/1 dilution) (Figure 6a, see also Supporting Information File 1, Figure S1). The lowest viability of Ch/CA-Ag NPs in the U-118 MG cell line was observed at 68.1 mg/L (1/1 dilution), and there were also no significant differences between 22.7 mg/L (1/2 dilution with *p* = 0.863) and 17.0 mg/L (1/3 dilution with *p* = 0.236) (Figure 6c). Consequently, for Ch/CA-Ag NPs, the cell viability of U-118 MG cell lines was 37% at 11.4 mg/L (1/5 dilution), while it reduced to 19% at 68.2 mg/L (1/1 dilution) (Figure 6c). When the effect of Ch/Q- and Ch/CA-Ag NPs in the U-118 MG cell line was compared at similar Ag concentrations, cell viability was found to be higher for Ch/Q-Ag NPs. For instance, cell viability was 33% at 13.8 mg/L (1/2 dilution) for quercetin, while it was 27% at 13.6 mg/L (1/4 dilution) for caffeic acid. Besides, cell viability was 59% at 10.4 mg/L (1/3 dilution) for quercetin, while it was 37% at 11.4 mg/L (1/5 dilution) for caffeic acid (Figure 6a and Figure 6c). These results show that the capping agent surrounding the silver nanoparticles have considerable dose-dependent effects against the cancer cell line (U-118 MG), and that the cell viability has constantly decreased by increasing the concentration of all synthesized nanoparticles. These findings are consistent with the results of other studies with silver and chitosan nanoparticles. For instance, Prema and Thangapandiyan synthesized and searched chitosan-stabilized Ag NPs for their cytotoxic effects against MCF-7 and HepG-2 cancer cell lines [57]. The results of the study showed an enhanced activity of chitosan-stabilized Ag NPs in comparison to colloidal Ag NPs, and the cytotoxicity was inversely proportional to the size of chitosan-stabilized Ag NPs, but directly proportional to their concentrations. In another recent study, antioxidants–chitosan–silver nanoparticles have been prepared and exhibited good and dose-dependent cytotoxicity against MCF-7 breast cancer cell lines [58].

When adding Ch/Q- and Ch/CA-Ag NPs to ARPE-19 cells, the viability values were also lower than those of the control group, especially for caffeic acid Ag NPs (Figure 6b and Figure 6d). When Ch/Q-Ag NPs doses were compared, the highest cell viabilities (95–98%) were observed at 6.9 mg/L (1/5 dilution) and 8.3 mg/L (1/4 dilution), respectively (Figure 6b). For Ch/Q-Ag NPs, the lowest cell viabilities (51–52%) were observed at 41.4 mg/L (1/1 dilution) and 13.8 mg/L (1/2 dilution), respectively, and there were no significant differences between these doses (*p* = 0.999). Besides, cell viabilities (73–89%) for 10.4 mg/L (1/3 dilution) and 8.3 mg/L (1/4 dilution) concentrations were found to be statistically significant (*p* = 0.002). Moreover, the viabilities of the ARPE-19 cells found for all concentrations of Ch/CA-Ag NPs were lower than the ARPE-19 cell viability in the control group according to the Dunnett t post hoc test results (all p-values were below 0.001 for all doses). The highest cell viability (31%) was observed at 11.4 mg/L (1/5 dilution), and this was higher than those all other doses (all p-values were below 0.001 for all doses). Conversely, the lowest viability (19%) was observed at 68.2 mg/L (1/1 dilution ratio), while there was no significant difference between 22.7 mg/L (1/2 dilution) (*p* = 0.976) and 17.0 mg/L (1/3 dilution) (*p* = 0.542), however, it was lower than those for concentrations of 13.6 mg/L (1/4 dilution) (*p* < 0.001) and 11.4 mg/L (1/5 dilution) (*p* < 0.001).

When Ch/Q- and Ch/CA-Ag NPs treatments against ARPE-19 cell lines were compared at similar Ag concentrations, cells viabilities were also found to be higher for quercetin NPs. For instance, cell viability was 52% at 13.8 mg/L (1/2 dilution) for quercetin, while it was 26% at 13.6 mg/L (1/4 dilution) for caffeic acid. Also, the cell viability was 73% at 10.4 mg/L (1/3 dilution) for quercetin, while it was 31% at 11.4 mg/L (1/5 dilution) for caffeic acid (Figure 6b and Figure 6d). Considering all the results for Ch/Q-Ag NPs, the cell viabilities of U-118 MG and ARPE-19 were 78% and 95% at 6.9 mg/L (1/5 dilution), while they reduced to 27% and 51% at 41.4 mg/L (1/1 dilution), respectively. On the other hand, for Ch/CA-Ag NPs, the cell viabilities of U-118 MG and ARPE-19 were 37% and 31% at 11.4 mg/L (1/5 dilution), even though it reduced to 19% at 68.2 mg/L (1/1 dilution) for both cells, respectively.

In summary, the concentration-dependent cell death in the U-118 MG human glioblastoma cells by Ch/Q-Ag NPs differs from cell death in the healthy ARPE-19 cells. The percentage of cell death caused by Ch/Q-Ag NPs is higher in U-118 MG cells than in ARPE-19 cells at the same concentrations. The quercetin-containing Ag nanoparticles (Ch/Q-Ag NPs) may be accepted as a candidate for use in cancer treatment. Furthermore, the concentration-dependent cell death caused by Ch/CA-Ag NPs in U-118 MG cells is comparable to that in the healthy ARPE-19 cells, suggesting that the effect may not be tissue specific.

Another issue that may affect the results of biological applications of NPs in general and should be considered is how the colloidal distribution and stability of the NPs are affected in the cell culture medium [74]. Since NPs are exposed to various forces that affect their stability and size in such environments (containing electrolytes, proteins and lipids), they may tend to collapse and aggregate. Although no aggregation and turbidity were observed during the preparation stage with the naked eye and in the microscope images of Ch/Q- and Ch/CA-Ag NPs in cell medium (see Supporting Information File 1, Figure S1), the stability of the synthesized NPs and how the size changes in cell culture medium compared to water were also evaluated by UV–vis and zeta potential measurements. An increase in the size of a metal nanoparticle gives rise to a decrease in the surface-to-volume ratio, which results in a decrease in the concentration of free surface electrons. Then, the required energy to polarize the electrons decreases, which leads to a redshift in the peak corresponding to the SPR absorption band. In other words, in the presence of the medium for cell culture (i.e., DMEM without phenol red), the possible formation of larger structures through the aggregation of Ag NPs will cause a shift in the position of the SPR peak toward the longer-wavelength side of the spectrum. Therefore, to get an insight into the aggregation and the stability of the Ch/Q- and Ch/CA-Ag NPs in the cell culture medium, UV–vis absorption measurements were carried out (see Supporting Information File 1, Figure S2). As can be seen in Figure S2 (Supporting Information File 1), the SPR peak emerges at 428 and 440 nm for Ch/Q- and Ch/CA-Ag NPs, respectively, in the cell culture medium. Compared with the NPs in water (see Figure 2b and Figure 2c), a redshift by about 11 and 16 nm of the SPR absorption peak for Ch/Q- and Ch/CA-Ag NPs, respectively, was found. In addition, a broadening was observed in the absorption curve for both Ag NPs in the medium. The redshift in the SPR peak wavelength and the broadening in the absorption curve towards the lower-energy region of the spectrum in the cell culture medium can be an indication of the formation of the aggregated structures [75]. UV–vis absorption measurements were also performed as a function of time to determine the stability of both Ch/Q- and Ch/CA-Ag NPs, which are highly stable in water, in the cell culture medium (see Figure S3, Supporting Information File 1). The measurements indicate that the absorbance of both Ag NPs decreases with time in the cell culture medium. This reveals the decreasing stability for both Ch/Q- and Ch/CA-Ag NPs in the cell culture medium compared to water, which can be explained by the formation of aggregated structures in the medium. In addition, the stability of the NPs was also determined by zeta potential measurements for both water and cell culture medium (with 1/10 dilution). The zeta potential values of Ch/Q- and Ch/CA-Ag NPs in water were found to be 37.1 ± 1.2 and 28.4 ± 2.2 mV, whereas they were −5.58 ± 0.47 and −2.08 ± 0.16 mV in medium (i.e., DMEM without phenol red), respectively. The variation in the zeta potentials, both regarding sign and numbers, clearly reveals that the stability of the NPs has changed significantly. Various studies have reported that in cell culture medium, there are pH-induced changes of NP conformation, particle size, stability, and functionality. Therefore, the nature of the cell culture medium should be kept in mind [74]. There are some approaches to provide electrostatic, steric, or electro-steric stabilization by adding electrolytes or polymeric substances to prevent aggregation of NPs [74]. However, in this study, no extra substance was added to the cell culture medium, and the direct interaction of Ag NPs with the cell lines was examined. In future studies, it will be valuable to examine the effect of additives that increase the stability of NPs and prevent their aggregation.

Moreover, the antibacterial activity of synthesized nanoparticles, Ch/Q- and Ch/CA-Ag NPs, were studied against Gram-negative (*P. aeruginosa* and *E. coli*) and Gram-positive (*S. aureus* and *S. epidermidis*) bacteria using the disc diffusion method. The inhibition zone diameters obtained after adding Ch/Q- and Ch/CA-Ag NPs in three different concentrations (1/1, 1/3, and 1/5 dilutions) are presented in Table 1.

**Table 1:** Antibacterial activity of Ch/Q- and Ch/CA-Ag NPs and standard antibiotics (ampicillin and amikacin) in terms of inhibition zone diameters by disc diffusion. Values in parentheses indicate the dilution ratios from the stock NPs and the measured diameters. A p-value of 0.05 was considered a statistical significance level.

inhibition zone diameter [mm]											
standard bacterial strains	Ch/Q-Ag NPs [mg/L]					Ch/CA-Ag NPs [mg/L]					
				
	41.4 (1/1)	10.4 (1/3)	6.9 (1/5)	*p*		68.2 (1/1)	17.0 (1/3)	11.4 (1/5)	*p*	ampicillin	amikacin

*P. aeruginosa*	9 (8–9)	7 (7–8)	7 (6–7)	0.047		9 (8–10)	7 (6–7)	6 (6–7)	0.050	—	16
*E. coli*	8 (7–8)	7 (6.5–7)	6 (6–6.5)	0.040		7 (7–8)	6 (6–6.5)	6 (5.5–6)	0.038	15	—
*S. epidermidis*	8 (8–9)	8 (7–8)	7 (7–7.5)	0.085		8 (8–9)	8 (7–8)	6 (6–7)	0.047	18	—
*S. aureus*	8 (8–9)	7 (7–7.5)	6 (6–7)	0.033		8 (8–9)	8 (7–8)	7 (6–7)	0.057	19	—

Almost all NPs are generally effective against both Gram-negative and Gram-positive bacteria. The median disc diameters of the Ch/Q- and Ch/CA-Ag NPs for all bacterial strains decreased as the dilution rate increased. According to the post hoc test results, these differences of Ch/Q-Ag NPs were statistically significant for *P. aeruginosa* (*p* = 0.047), *E. coli* (*p* = 0.040), and *S. aureus* (*p* = 0.033), but not for *S. epidermidis* (*p* = 0.085). The median disc diameters of Ch/CA-Ag NPs were statistically significant for *P. aeruginosa* (*p* = 0.050), *E. coli* (*p* = 0.038), and *S. epidermidis* (*p* = 0.047), but not for *S. aureus* (*p* = 0.057). A graphical evaluation of Table 1 is given in Figure S3 (Supporting Information File 1). In this study, inhibition zones of control antibiotics (ampicillin and amikacin) were also evaluated according to the European committee on antimicrobial susceptibility testing (EUCAST) (see section “Antibacterial activity”). In comparison with ampicillin and amikacin, the median disc diameters of the NPs were generally lower (Table 1). These findings can be compared or correlated with the results of some recent antibacterial studies. For instance, in a study by Alavi et al., quercetin–Ag NPs of similar size (11 nm) displayed good inhibition against *P. aeruginosa* and *S. aureus* at 2 and 4 μg/mL [76]. In another comparative antibacterial study, quercetin-loaded and unloaded Ag NPs did not exhibit any inhibition against the Gram-positive bacterium *S. aureus* [77]. While unloaded Ag NPs exhibited inhibition against the Gram-negative bacterium *E. coli* at low concentrations (1–2 µg/mL), quercetin loaded-Ag NPs showed no antibacterial effect. In contrast to these finding, quercetin-decorated Ag NPs at concentrations of 5 and 10 µg/mL exhibited stronger antibacterial and antibiofilm activities against multidrug-resistant *E. coli* strains when compared to Ag NPs or quercetin treatments [78]. Here, factors such as low NP concentrations, high bacterial concentration, nanoparticle size, and ambient pH may have played a role in obtaining such low antibacterial inhibition results. Furthermore, it may be possible that the NPs could not diffuse from the impregnated disc to the agar medium compared to a chemical antibacterial agent.

## Conclusion

In the present study, the one-pot synthesis of quercetin and caffeic acid-functionalized chitosan-capped colloidal silver nanoparticles (Ch/Q- and Ch/CA-Ag NPs) was successfully performed. The characterization of Ch/Q- and Ch/CA-Ag NPs was done by using UV–vis, FTIR, and TEM measurements. The characteristic surface plasmon resonance (SPR) absorption bands at 404 nm for Ch-Ag NPs (with chitosan), shifted to 417 and 424 nm for Ch/Q- (with quercetin) and Ch/CA-Ag NPs (with caffeic acid), respectively. Besides, new shoulders at lower wavelengths in the obtained spectra were the indication for the presence of quercetin and caffeic acid in Ch/Q- and Ch/CA-Ag NPs. The observed broadening in the absorption spectra was attributed to the increased size and shape distribution with these co-capping agents. TEM images revealed that both Ch/Q- and Ch/CA-Ag NPs were spherical. When the size distributions were examined, the mean particle size of Ch/Q-Ag NPs was 11.2 ± 2.4 nm, and that of Ch/Q-Ag NPs was 10.3 ± 2.4 nm. Furthermore, TEM images of Ch/Q-Ag NPs after negative staining were obtained and confirmed that the chitosan/quercetin shell structure covered the core Ag NPs. Moreover, the amount of quercetin and caffeic acid in the samples of Ch/Q- and Ch/CA-Ag NPs determined by colorimetric methods were found to be 31.0 ± 0.8 and 28.8 ± 0.4 μg/mL, respectively. The anticancer properties of Ch/Q- and Ch/CA-Ag NPs at different concentrations were investigated against U-118 MG human glioblastoma and ARPE-19 human retinal pigment epithelium cells. The results showed that the cell viability values for U-118 MG and ARPE-19 were 78% and 95% at 6.9 mg/L, while they reduced to 27% and 51% at 41.4 mg/L, for Ch/Q-Ag NPs, respectively. The cell viability values for Ch/CA-Ag NPs for U-118 MG and ARPE-19 cell lines were 37% and 31% at 11.4 mg/L, whereas they reduced to 19% at 68.2 mg/L, respectively. Accordingly, due to the cancer-specific effect of Ch/Q-Ag NPs, quercetin-containing NPs may be a candidate for cancer treatment in the future. In addition, an antibacterial evaluation of Ch/Q- and Ch/CA-Ag NPs against Gram-negative (*P. aeruginosa* and *E. coli*) and Gram-positive (*S. aureus* and *S. epidermidis*) bacteria was performed. The NPs were effective against both Gram-negative bacteria and Gram-positive bacteria. More specifically, Ch/Q- and Ch/CA-Ag NPs exhibited a bit higher inhibitions against *P. aeruginosa*. Consequently, such combinations may provide several advantages in targeted drug delivery systems, such as minimal toxicity to normal cells and enhanced/synergistic effects against cancer cells. Moreover, this combination approach with different natural products in binary or ternary systems will offer a better synergistic effect against various bacteria or fungi. According to the results of this preliminary study, chitosan-stabilized silver nanoparticles containing caffeic acid or quercetin may have therapeutic effects against U-118 MG cancer cell lines and against some pathogenic bacteria. In vivo models and clinical trials will be necessary to understand the localization of nanoparticles inside cells and the mode of action.

## Supporting Information

File 1 Additional figures.
