# Supplementary material for: Quercetin- and caffeic acid-functionalized chitosan-capped colloidal silver nanoparticles: one-pot synthesis, characterization, and anticancer and antibacterial activities
Source: Beilstein J Nanotechnol. 2023 Mar 20;14:362–76. doi: 10.3762/bjnano.14.31 (PMC10043739; doi:10.3762/bjnano.14.31)
Supplement: File 1 — Additional figures. [file Beilstein_J_Nanotechnol-14-362-s001.pdf]

BEILSTEIN JOURNAL OF NANOTECHNOLOGY

Supporting Information

for

Quercetin- and caffeic acid-functionalized chitosan-capped colloidal silver nanoparticles: one-pot synthesis, characterization, and anticancer and antibacterial activities

Akif Hakan Kurt, Elif Berna Olutas, Fatma Avcioglu, Hamza Karakuş, Mehmet Ali Sungur, Cansu Kara Oztabag and Muhammet Yıldırım

Beilstein J. Nanotechnol. **2023,** *14,* 362–376. doi:10.3762/bjnano.14.31

Additional figures

License and Terms: This is a supporting information file under the terms of the Creative Commons Attribution License (https://creativecommons.org/licenses/by/4.0). Please note that the reuse, redistribution and reproduction in particular requires that the author(s) and source are credited and that individual graphics may be subject to special legal provisions.
The license is subject to the *Beilstein Journal of Nanotechnology* terms and conditions: (https://www.beilstein-journals.org/bjnano/terms)

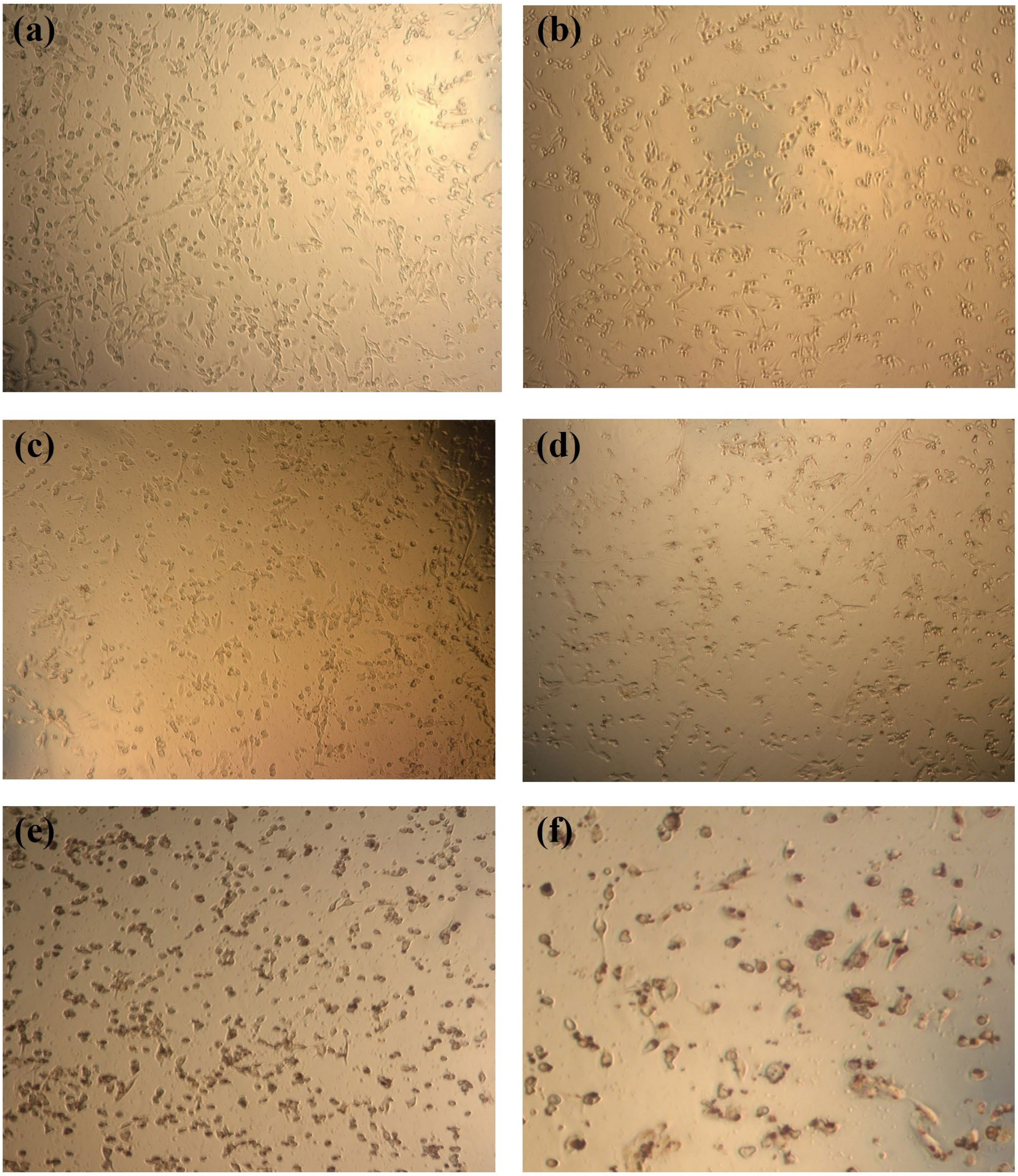

Figure S1: Morphological images of the U118-MG cell line (a) in medium only (no NPs), after applying different concentrations of Ch/Q-Ag NPs in dilutions of (b) 1/5, (c) 1/4, (d) 1/3, (e) 1/2, and (f) 1/1.

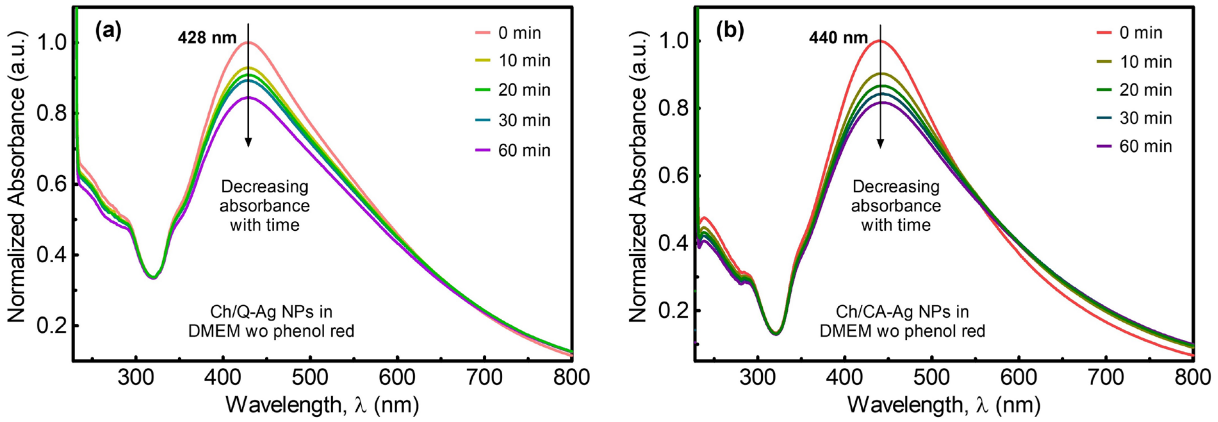

Figure S2: Time-dependent UV–vis absorption measurements for (a) chitosan/quercetin (Ch/Q-)- and (b) chitosan/caffeic acid (Ch/CA-)-capped Ag NPs in DMEM medium without phenol red (1/10 dilution).

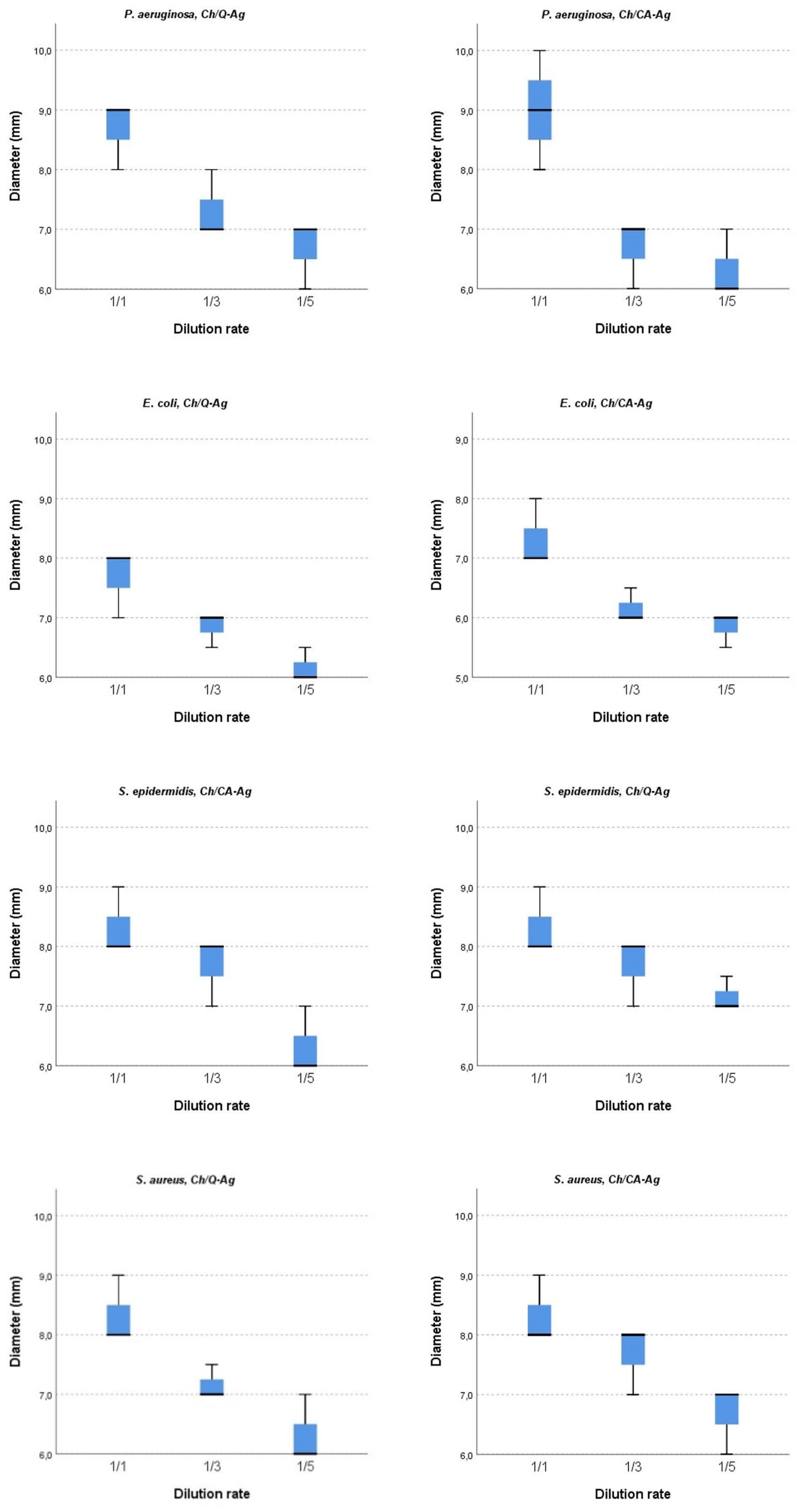

Figure S3: Antibacterial activities of Ch/Q- and Ch/CA-Ag NPs in terms of inhibition zone diameters by disc diffusion. Graphical evaluation of Table 1 in the article.
